# An Efficient Binary Sand Cat Swarm Optimization for Feature Selection in High-Dimensional Biomedical Data

**DOI:** 10.3390/bioengineering10101123

**Published:** 2023-09-25

**Authors:** Elnaz Pashaei

**Affiliations:** 1 Department of Computer Engineering, Istanbul Aydin University, Istanbul 34295, Turkey; elnazpashaei@aydin.edu.tr; 2 Department of Medical and Molecular Genetics, Indiana University School of Medicine, Indianapolis, IN 46202, USA

**Keywords:** sand cat swarm optimization, pinhole-imaging-based learning, feature selection, biomedical data, cancer prediction

## Abstract

Recent breakthroughs are making a significant contribution to big data in biomedicine which are anticipated to assist in disease diagnosis and patient care management. To obtain relevant information from this data, effective administration and analysis are required. One of the major challenges associated with biomedical data analysis is the so-called “curse of dimensionality”. For this issue, a new version of Binary Sand Cat Swarm Optimization (called PILC-BSCSO), incorporating a pinhole-imaging-based learning strategy and crossover operator, is presented for selecting the most informative features. First, the crossover operator is used to strengthen the search capability of BSCSO. Second, the pinhole-imaging learning strategy is utilized to effectively increase exploration capacity while avoiding premature convergence. The Support Vector Machine (SVM) classifier with a linear kernel is used to assess classification accuracy. The experimental results show that the PILC-BSCSO algorithm beats 11 cutting-edge techniques in terms of classification accuracy and the number of selected features using three public medical datasets. Moreover, PILC-BSCSO achieves a classification accuracy of 100% for colon cancer, which is difficult to classify accurately, based on just 10 genes. A real Liver Hepatocellular Carcinoma (TCGA-HCC) data set was also used to further evaluate the effectiveness of the PILC-BSCSO approach. PILC-BSCSO identifies a subset of five marker genes, including prognostic biomarkers HMMR, CHST4, and COL15A1, that have excellent predictive potential for liver cancer using TCGA data.

## 1. Introduction

Enormously large, rapidly growing collections of biomedical and clinical data pose significant challenges to their analysis and interpretation. Health data are large-scale, multimodal, and high-dimensional. The promise of Big Data in healthcare is based on the ability to discover patterns and transform massive volumes of data into meaningful information for precision, diagnosis, treatment, and decision-makers. Biomedical datasets, encompassing genomics, proteomics, clinical attributes, imaging, and more, often present researchers with a staggering number of variables. While this wealth of data holds the potential to unveil crucial insights into disease mechanisms and patient profiles, it simultaneously poses formidable challenges, giving rise to the ‘curse of dimensionality’.

In biomedical data analysis, the ‘curse of dimensionality’ arises from the combination of high-dimensional feature spaces, sparsity, computational demands, risk of overfitting, and the need to capture complex biological phenomena. Addressing this challenge requires innovative feature selection techniques and dimensionality reduction methods. This difficulty in navigating high-dimensional biomedical data has led to a growing interest among researchers in the biomedical domain, inspiring the development of new robust algorithms that are best suited to appropriately evaluate this big data [1]. The task of extracting meaningful information and identifying key aspects within these vast datasets has become a focal point of exploration and innovation within the field of biomedical research.

Feature selection is a powerful data mining approach for shrinking the dimensionality of feature space. It is broadly known that feature selection is an NP-hard task, and therefore determining the optimal or near-optimal feature set is a challenging task [2,3].

Feature selection’s primary role is to identify and retain the most informative and relevant attributes while discarding redundant or noisy variables. Doing so not only mitigates the computational burden associated with high dimensionality but also enhances the interpretability and generalization of analytical models. In the context of disease diagnosis, feature selection serves as a compass guiding researchers and clinicians toward the most discriminating biomarkers or attributes associated with specific diseases. This precision enables the development of diagnostic models that are not only accurate but also clinically interpretable. Such models, informed by selected features, provide the foundation for early disease detection and stratification, facilitating timely interventions and improved patient outcomes. Moreover, feature selection plays a pivotal role in patient care management. In the era of personalized medicine, where treatment strategies are tailored to individual patients, the identification of relevant biomarkers and clinical attributes is paramount. Feature selection aids in constructing predictive models that inform treatment decisions, predict patient responses, and gauge disease prognosis. By focusing on the most influential factors, healthcare providers can optimize treatment plans, minimize adverse effects, and maximize therapeutic efficacy.

There are three popular feature selection methods: filter-based, wrapper-based, and hybrid approaches. Filter techniques assess the importance of features based on their correlation with the dependent variable using statistical methods and are significantly quicker than wrapper approaches, whereas wrapper methods assess the utility of a subset of features by training a model on it and can provide the most effective subset of features. Nature-inspired optimization algorithms (NIOAs) are used as search techniques in wrapper methods to identify informative features. A hybrid feature selection method combines filters and wrappers approaches. Hybrid approaches are still in their fancy and further research is needed to develop a more effective feature selection methodology [4]. In the literature, various feature selection strategies have been offered. Some of them are a hybrid of minimum redundancy maximum relevance (mRMR) and a mutated binary Aquila optimizer (MBAO) [5], a hybrid of mutual information maximization (MIM) and moth flame optimization algorithm (MFOA) [6], binary coral reefs optimization with simulated annealing and tournament selection strategy (BCROSAT) [1], an improved binary clonal flower pollination algorithm (IBCFPA) [7], an improved shuffled frog leaping algorithm (ISFLA) [8], a hybrid of mRMR with a combination of binary black hole algorithm and binary dragonfly optimization algorithm (DBH) [9], a hybrid of symmetrical uncertainty (SU) and reference set harmony search algorithm (RSHSA) [10], “Technique for Order Preference by Similarity to Ideal Solution” (TOPSIS) filtering and binary Jaya algorithm [11], a hybrid of information gain (IG) and modified krill herd algorithm (MKHA) [12], and hybrid of mRMR and binary Coot with simulated annealing and crossover operator (mRMR BCOOT-CSA) [13]. Difficulty in parameter tuning, lack of interpretability, risk of premature convergence, and limited adaptability are some limitations of the above approaches. Nevertheless, recognizing that no single solution can entirely alleviate the dimensionality curse within the original dataset, these limitations have motivated numerous researchers to propose new algorithms with the aim of achieving improved performance. 

The sand cat swarm optimization (SCSO) algorithm [14] is a new NIOA, that has been utilized to solve various optimization problems such as engineering problems [15,16], power transformer fault diagnosis [17], and feature selection [2,18]. Low solution precision and early convergence are two main drawbacks of most existing SCSO variations [15]. This paper puts forward an improved version of binary SCSO (PILC-BSCSO) by incorporating crossover and opposition-based learning for feature selection challenges of high-dimensional medical data. This is the main innovation of this paper and shows promise in finding the best feature subset. 

The key contributions of this paper are as follows:

- A novel gene selection approach is proposed based on an enhanced binary sand cat swarm optimization for high-dimensional biomedical data.
- A pinhole-imaging opposition-based learning (PIOBL) scheme is employed to boost the exploration and convergence characteristics of the BSCSO.
- The Crossover operator is fused with BSCSO to improve the search performance of the original BSCSO.
- An initial population strategy based on the Differential Expression (DE) analysis is conducted to identify differentially expressed genes (DEGs), which makes the proposed algorithm, called PILC-BSCSO, obtain higher classification accuracy with a better-initialized population.
- The suggested PILC-BSCSO approach is compared to 11 state-of-the-art methods on three benchmark microarray datasets and outperforms them all.
- The efficiency of the PILC-BSCSO approach was further assessed using a real Liver Hepatocellular Carcinoma (TCGA-HCC) data set, and PILC-BSCSO selects a subset of five marker genes while offering the best accuracy.

## 2. Materials and Methods

### 2.1. Sand Cat Swarm Optimization

The SCSO Algorithm is a new nature-inspired optimization algorithm proposed by Seyyedabbasi [14], which simulates the behavior of sand cats in hunting. These animals utilize their acute hearing to detect low-frequency disturbances. Therefore, they may sense prey movement underground. They also have an unusual ability to dig swiftly if the prey is underground. In SCSO, the population consists of N sand cat individuals (solutions) with D dimensions, thus the population vector contains an N×D dimensional matrix. The X(t) demonstrates the position vector of each sand cat in searching space at iteration t. 

The sound cat has a sensitivity range of (2, 0) kHz in perceiving low-frequency noises. It starts at 2 kHz and decreases linearly till it approaches 0 kHz. The sensitivity level is known as rg in SCSO, which is calculated as follows:

(1) $$rg=s_{M}-\left(\frac{s_{M}\times t}{T}\right)$$

where $s_{M}$ is taken to be 2. *t* is the current iteration number, while *T* is the maximum number of iterations. Meanwhile, the *R* parameter determines the trade-off between the exploration and exploitation phases and is computed as follows:

(2) $$R=((2\times rg)\times rand\left(0,\;1\right))-rg$$

where $rand\left(0,\;1\right)$ produces a random number between 0 and 1. The r parameter, which specifies the sensitivity range of each potential solution, is determined as follows:

(3) $$r=rg\times rand\left(0,\;1\right)$$

The sand cat’s next location is decided by the value of R, which runs between −1 and 1. When $\left|R\right|\leq 1$, the SCSO approach concentrates on exploitation and guiding the sand cat to hunt the prey (4–5). Otherwise, the algorithm concentrates on exploration and forces the sand cats to look for food (6–8).

In SCSO the mathematical expression of attacking the prey (exploitation) is as follows:

(4) $$X_{rand}=\left|rand\left(0,\;1\right)\times X_{best}-X(t)\right|$$

(5) $$X_{\left(t+1\right)}=X_{best}-rand\left(0,\;1\right)*X_{rand}*\cos\;(\theta )$$

where $X_{rand}$ calculates the distance between the best position $X_{best}$ and current position X(t) in the related iteration t. $X_{\left(t+1\right)}$ demonstrates the position update for the corresponding search agent, i.e., X. Moreover, the sand cats’ precise sensitivity is supposed to be circular, hence the direction of each movement is decided by a random angle θ based on a roulette wheel selection.

In SCSO, the mathematical expression of searching for prey (exploration), is as follows:

(6) $$cp=floor\left(N*rand(0,\;1)+1\right)$$

(7) $$X_{Candidate}\left(t\right)=X(cp,:)$$

(8) $$X_{\left(t+1\right)}=r\times (X_{Candidate}\left(t\right)-rand\left(0,\;1\right)\times X(t))$$

where $X_{Candidate}\left(t\right)$ indicates a random candidate position. The pseudo-code of the SCSO algorithm is shown in Algorithm 1.
**Algorithm 1:** Pseudo-code of the SCSO algorithm.1.Determine the number of population N, and maximum number of iteration T
2.Initialize the sand cat population $X_{i}\left(i=1,\;2,\;\ldots ,\;N\right)$
3.**While** t≤T**do**4. Calculate the fitness function of each sand cat based on the objective function5. Determine $X_{best}$
6. Calculate rg when $s_{M}=2$
7. **For** i=1 **to** N **do**8.  Calculate R and r
9.  **For** j=1 **to** D **do**10.   Randomly selected 0≤θ≤360 using Roulette wheel selection11.   **if**
((−1<=R)&&(R<=1)) **then**12.    $X_{rand}=\left|rand\left(0,\;1\right)\times X_{best,j}-X_{\left(i,j\right)}\right|$
13.    $X_{\left(i,j\right)}=X_{best,j}-rand\left(0,\;1\right)*X_{rand}*\cos\;(\theta )$    //update position using (5)14.   
**else**
15.    
$cp=floor\left(N*rand(0,\;1)+1\right)$
16.    
$X_{Candidate}=X(cp,:)$
17.    $X_{\left(i,j\right)}=r\times (X_{Candidate,j}-rand\left(0,\;1\right)\times X_{\left(i,j\right)})$//update position using (8)18.   
**End if**
19.  
**End for**
20. 
**End for**
21. 
t=t+1
22.**End while**23.**Return** $X_{best}$

### 2.2. Binary Sand Cat Swarm Optimization for Feature Selectıon

In the context of feature selection, each feature can be thought of as a binary decision–either included in the final subset or not. This binary choice can be represented using a binary vector of size D, where D is the total number of features in the dataset. Each element of the vector corresponds to a feature, and is set to 1 if the feature is selected and 0 if not.

The SCSO method is applied in a continuous space, whereas the feature selection problem is applied in a discrete space. Before the SCSO algorithm can be used for the feature selection issue, the continuous space must be transformed into the discrete space. The transfer functions are used for this conversion. Seyyedabbasi [18] presented the first binary version of the SCSO method, which employed a V-shaped transfer function. The transfer function determines the probability that the binary solution element changes from 0 to 1. Also, Qtaish et al. [2] introduced a memory-based BSCSO (BMSCSO) method that incorporates a memory-based approach into the BSCSO position-updating process, employing an S-shaped transfer function to pick the most relevant subset of features. 

### 2.3. Pinhole Imaging Opposition-Based Learning

**Figure 1:**
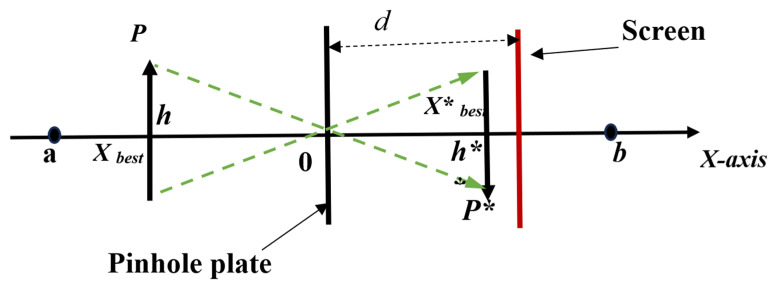
Principle of pinhole imaging opposition-based learning.

Various techniques, including mutation [5], Lévy flight [19], and opposition-based learning (OBL) [20], have been used in the literature to increase NIOA’s exploration capabilities. OBL broadens the search range by computing the inverse of the existing viable solution and locating candidate solutions in more ideal places. OBL is a subset of pinhole-imaging opposition-based learning (PIOBL) [21]. Pinhole imaging is a general physical phenomenon in which a light source flows through a tiny hole in a plate, forming an inverted actual picture on the opposite side of the plate. Figure 1 depicts the basic PIOBL concept.

The coordinate *x*-axis’ upper and lower bounds are labeled a and b in the picture. A tiny aperture screen is installed at the base point O. Once the light source via the small aperture receives a reversed image $p^{*}$ of height $h^{*}$ at the imaging screen, the projection of $p^{*}$ on the *x*-axis is ${X\;}_{best}^{*}$ (the newly created reverse solution), whereas the projection of *p* whose height is *h*, on the *x*-axis is $X_{best}$ (the current global optimal solution). The geometric connection of the line subdivisions in the figure allows us to deduce:

(9) $$\frac{\frac{\left(a+b\right)}{2}-X_{best}}{X_{best}^{*}-(a+b)/2}=\frac{h}{h^{*}}$$

Substituting $h/h^{*}=K$ into the foregoing equation produces the expression for $X_{best}^{*}$:

(10) $$X_{best}^{*}=\frac{\left(a+b\right)}{2}+\frac{\left(a+b\right)}{2K}-\frac{X_{best}}{K}$$

When the method is solving a high-dimensional complex function, $X_{best}^{*}$ can be computed using the following equation:

(11) $$X_{best,j}^{*}=\frac{\left(a_{j}+b_{j}\right)}{2}+\frac{\left(a_{j}+b_{j}\right)}{2K}-\frac{X_{best,j}}{K}$$

where $X_{best,j}^{*}$ is the inverse solution of $X_{best,j}$, and $X_{best,j}$ demonstrates the optimal solution in the jth dimension. $a_{j}$ and $b_{j}$ are the minimum and maximum values in the jth dimension and the scale factor K=0.05.

### 2.4. Single Point Crossover

Crossover is a genetic operator that mixes two parents’ genetic information to produce new offspring. After selecting a random cut point on parents to create offspring, all data in the parents’ string after that point is swapped between the two parents.

### 2.5. The Proposed Algorithm

A modified binary SCSO (called PILC-BSCSO) with pinhole-imaging-based learning and crossover operator is proposed as a novel wrapper feature selection to find the optimal gene subset with the highest accuracy. 

The crossover operator is a fundamental mechanism in BSCSO, facilitating the exchange of genetic information to create diverse offspring. This diversity enhances the algorithm’s search capabilities, allowing it to effectively explore a wider range of feature combinations and identify feature subsets with improved predictive power for biomedical data analysis.

The pinhole-imaging-based learning strategy provides a localized focus as well as adaptability and balance in the BSCSO process. It strategically narrows the focus when needed for in-depth exploration and widens it to exploit promising regions. This intelligent strategy not only enhances the algorithm’s ability to navigate the vast solution space but also safeguards against premature convergence, ultimately contributing to its effectiveness in feature selection for high-dimensional biomedical data analysis.

The detailed implementation of the proposed algorithm is elaborated upon in the following steps:

Step 1. First, a Limma differential expression analysis of microarray data is conducted as a preprocessing step to identify DEGs, and the genes with an adjusted *p*-value lower than 0.05 are selected. Then, the shrink dataset (GEGs) is used as the input for the proposed PILC-BSCSO algorithm where the Cohen’s kappa score of the support vector machine (SVM) [22,23,24] with the linear kernel is utilized as the fitness function. 

Step 2. Population initialization is performed, and each sand cat individual is encoded as a binary vector with an initial value of 1. 

Step 3. Binary SCSO is used to further select the optimal subset of genes from a provided pool of DEGs. Each individual within the sand cat population undergoes fitness value computation, enabling the identification of the individual with the most optimal fitness—a role granted to the best individual. After this process, the updating of the solution is performed using (5) and (8). The transfer function affects the efficiency of binary optimization techniques. There are several transfer functions accessible in the literature; nevertheless, selecting one is not an easy process [25]. We are using a hyperbolic tangent sigmoid (tansig) transfer function to convert the continuous SCSO algorithm to a binary version with the following equations:

(12) $$Tf\left(X_{\left(i,j\right)}\right)=\frac{2}{1+e^{-2*X_{\left(i,j\right)}}}-1$$

(13) $$X_{\left(i,j\right)}=\left\{\begin{array}{l} 1,\;\;\;Tf(X_{\left(i,j\right)})>rand(0,\;1)\\ 0,\;\;\;otherwise \end{array}\right.$$

**Figure 2:**
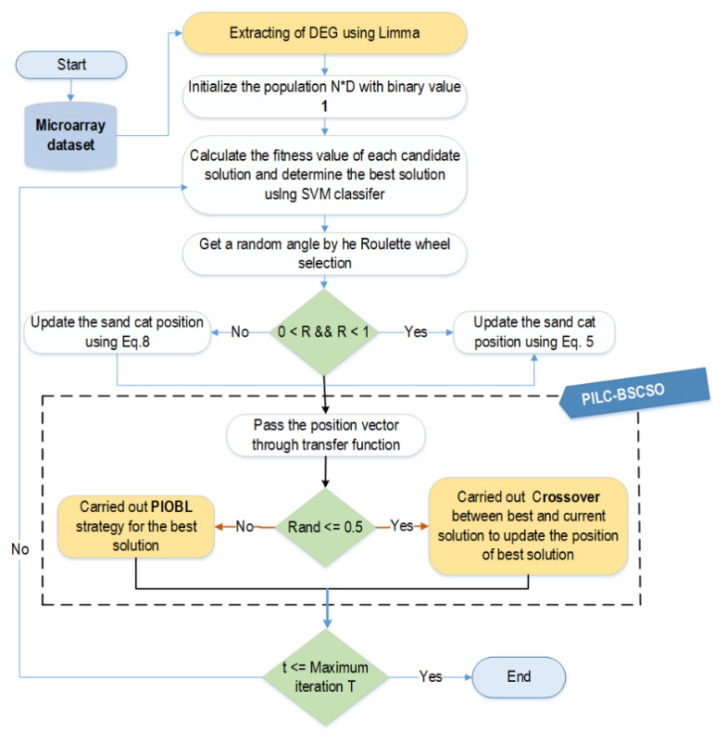
Flow chart of the proposed PILC-BSCSO algorithm for gene selection.

Step 4. Low solution accuracy and early convergence are two main drawbacks in the majority of current SCSO versions. Therefore, PIOBL and crossover mechanisms are utilized to effectively boost the exploration ability of SCSO. The process of updating individuals after step 3 is continued using either the crossover operators or the PIOBL strategy according to random probability. The individual updating procedure is repeated until the stop criteria are met. The comprehensive sequence of steps involved in the PILC-BSCSO algorithm is depicted in Figure 2, while the precise algorithmic details are provided in Algorithm 2.
**Algorithm 2:** Pseudo-code of the proposed PILC-BSCSO algorithm for feature selection.1.Load Microarray dataset2.Extracting DEG lists using Limma and obtaining shrinking dataset with D features3.//Perform PILC-BSCSO algorithm4.Determine the number of population N, and maximum number of iterations T5.$\mathrm{Initialize}\;\mathrm{the}\;\mathrm{sand}\;\mathrm{cat}\;\mathrm{population}\;X_{i}\left(i=1,\;2,\;\ldots ,\;N\right)$ with the binary value 16.**While** 
t≤T
**do**
7.    Calculate the fitness function of each sand cat using SVM with a 10-fold CV8.    $\mathrm{Determine}\;X_{best}$
9.    Calculate rg when $s_{M}=1$
10.    **For**
i=1 to N **do**11.        Calculate R
 and r
12.        **For**
j=1 to D **do**13.            Randomly selected 0≤θ≤360 using Roulette wheel selection14.            **if** ((0<R)&&(R<1)) **then**15.                Update the search agent position using Equation (5)16.            **else**
17.                Update the search agent position using Equation (8)18.            **End if**
19            $Tf=\frac{2}{1+e^{-2X(i,j)}}-1$
20            **if**
tf>rand(0, 1) **then** $X_{\left(i,j\right)}=1$ **else** $X_{\left(i,j\right)}=0$21.        **End for**//j22.        **if**
$rand\left(0,\;1\right)<0.5$ **then**23.            //Perform crossover operator24.            [q1, q2] = Crossover ($X_{best}$, $X_{\left(i,:\right)}$)25.            Calculate the fitness values of p1, p2 using SVM26.            **if** fitness value of q1 is better than fitness values of q2 and $X_{best}$ **then**27.                
$X_{best}=q1$
28.            **else if** th**e** fitness value of q2 is better than the fitness value of $X_{best}$ **then**
29.                $X_{best}=q2$
30.            
**End if**
31.        **else**
32.            //Perform PIOBL operator33.            $\mathrm{Calculate}\;q1=\frac{1}{2}+\frac{1}{2K}-\frac{X_{\left(i,\;:\right)}}{K}$ when k=0.05
34.            $X_{best}^{*}=q1\;AND\;X_{best}$
35.            Calculate the fitness values of $X_{best}^{*}$ using SVM36.            **if** the fitness value of $X_{best}^{*}$ is better than the fitness values of $X_{best}$ **then**37.                $X_{best}=X_{best}^{*}$
38.                $X_{\left(i,:\right)}$ = $X_{best}^{*}$
            **End if**

        **End if**
39.    **End for**//i40.    t=t+1
41.**End while**42.**Return** 
$X_{best}$


## 3. Results

### 3.1. Experimental Setup

The proposed method is a two-step procedure. In the first step, Z-score normalization and DEG analysis are performed as a preprocessing step to scale and identify genes whose expression levels differ significantly between the two experimental conditions. In the second step, the proposed approach is applied to gain an optimal subset of genes. The effectiveness of our proposed gene selection approach was examined on three binary-class microarray cancer datasets and one real The Cancer Genome Atlas Liver Hepatocellular Carcinoma (TCGA-LIHC) dataset. Table 1 describes the characteristics of the datasets. In this study, we employed an SVM classifier with a linear kernel as a fitness function to explore the prediction ability of gene subsets. Tuning parameter ‘C’ was held constant at a value of 1 (default value).

To avoid bias, we subjected each subset of potential candidate genes to rigorous validation and analysis, employing a repeated 10-fold cross-validation approach with three repetitions. To show stability, the proposed methodology was executed independently multiple times on distinct datasets, with subsequent reporting of the averaged outcomes. For the implementation of algorithms, the R programming language was used. Specifically, the ‘limma’ package was harnessed for the analysis of DEGs, while the construction of the SVM classifier was carried out using the ‘e1071’ package. The “rmcfs” package was used for Monte Carlo Feature Selection (MCFS) [26], while the “praznik” package was employed for feature ranking using Minimum Redundancy Maximum Relevance (mRMR) [27]. Particle Swarm Optimization (PSO) and Genetic Algorithms (GA) optimization techniques were implemented using the Weka platform. R code of PILC-BSCSO is available at https://github.com/nazpashaei/PILC-BSCSO, accessed on 27 August 2023.

Computational experiments were conducted on an AMD Ryzen 7 5700U processor operating at 1.80 GHz, ×64 architecture, and bolstered by 16 GB of RAM. For four optimization algorithms, we configured the algorithm parameters, setting the number of populations at 100 and the maximum number of iterations at 50.

### 3.2. Experimental Results on Three Benchmark Microarray Datasets

The results of this study reveal significant insights into the performance and effectiveness of the proposed approach. The investigation of Differentially Expressed Genes (DEGs) led to the identification of distinct gene sets across different datasets. Specifically, there are 358 DEGs with an adjusted *p*-value of 0.05 in the colon, 328 with a *p*-value of 0.05 in the CNS, and 154 with a *p*-value of 0.05 and |LogFC| > 0.68 in the Breast datasets, respectively. To evaluate the potential of these gene sets for classification tasks, the LOOCV (Leave-One-Out Cross-Validation) classification accuracy was assessed using an SVM classifier. mRMR and MCFS feature ranking algorithms with various cut-offs were utilized to compare with DEG performance. The mRMR is an entropy-based feature selection method that calculates the mutual information (MI) between a group of features and a class variable. Features with high MI values with respect to the class variable and low MI values with respect to other selected features are considered more informative and less redundant. The MCFS method evaluates the feature importance by creating numerous decision trees. Each decision tree is trained on a subset of the data with a random feature subset. The importance of each feature is determined by how much it contributes to the quality of the decision trees.

The outcomes, detailed in Table 2, provided an initial assessment of the DEGs’ predictive power compared to MRMR and MCFS. Table 2 reveals that MCFS with cutoffs of 100, 200, and 300 consistently demonstrates better classification accuracy on three datasets. Notably, the 300-cutoff threshold outperforms DEGs in terms of classification accuracy.

**Figure 3:**
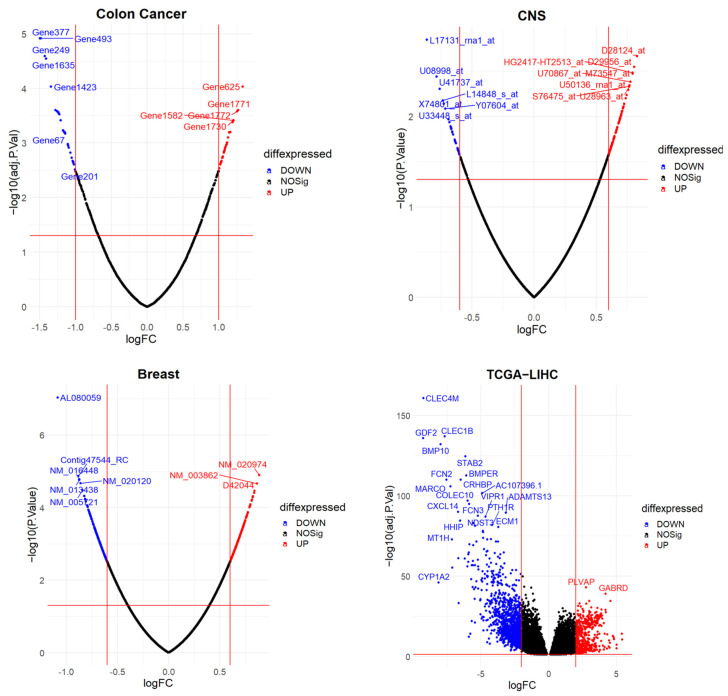
Volcano plot of the DEGs identified by limma for each dataset.

Visual representations further enhanced our understanding of the data. The volcano plot (Figure 3) depicted the distribution of Log2(fold-change) against the significance (*p*-value) of the identified DEGs, with cut-off values indicated by vertical and horizontal dotted lines. The comparison of the proposed PILC-BSCSO method with the basic BSCSO technique, PSO, and GA (Table 3) showcases their respective performance in 10 separate runs. Strikingly, PILC-BSCSO consistently outperformed all three swarm optimization algorithms (BSCSO, GA, and PSO) in terms of classification accuracy across all datasets. A nuanced observation was made for the colon and breast datasets, where BSCSO exhibited a slight advantage over PILC-BSCSO in terms of the average number of selected genes. Table 3 also shows the statistical test results, where a *p*-value < 0.05 indicates that the PILC-BCSO methodology produces statistically different results than other techniques.

**Figure 4:**
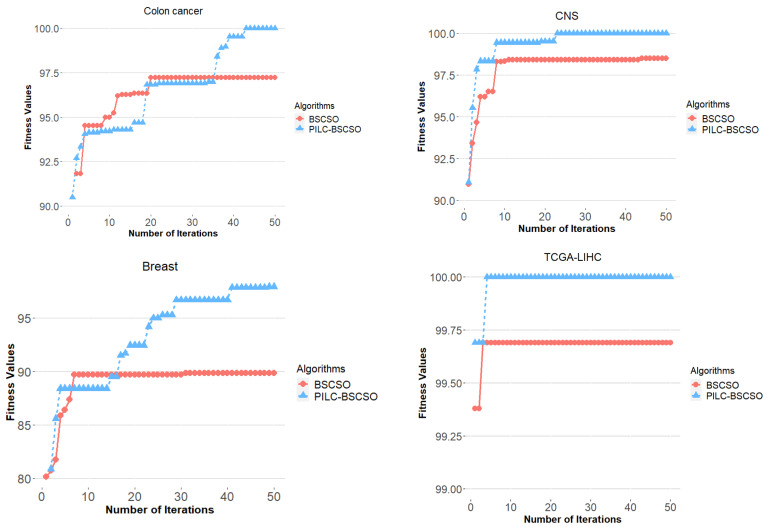
The convergence behavior of BSCSO and PILC-BSCSO for three microarray datasets.

The convergence behavior of PILC-BSCSO and the basic BSCSO methods was examined, and the results are depicted in Figure 4. This visualization showcases the trajectories of their convergence across four distinct datasets, all derived from the same random seed. Significantly, PILC-BSCSO exhibited more favorable convergence trends in terms of fitness value (Cohen’s kappa) compared to conventional BSCSO, which tended to converge to local optima. It is worth noting that PILC-BSCSO may take longer (two and a half times) to converge than the traditional BSCSO approach.

**Figure 5:**
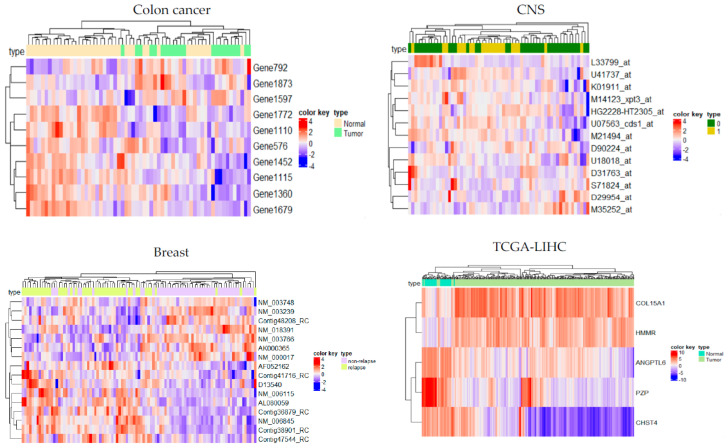
The gene expression level of the best subset of genes with the highest accuracy is shown as a heatmap.

Figure 5 offered a visual representation of the gene expression profiles for the best subset of discriminative genes identified by the proposed method for each dataset, represented through a heatmap.

**Figure 6:**
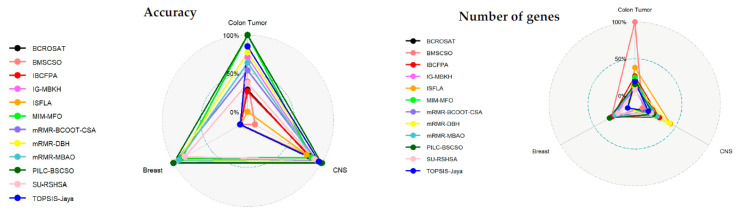
Comparing the performance of the suggested methodology to approaches from the literature.

To comprehensively assess the proposed method’s efficacy, comparisons were made against 11 state-of-the-art approaches. The average results, summarized in Table 4 and Figure 6, demonstrated that PILC-BSCSO consistently achieved superb classification accuracy while selecting a reasonable number of genes, outperforming 11 competing techniques across all three datasets. These findings collectively underscore the effectiveness of the proposed PILC-BSCSO approach in identifying significant gene subsets and its potential for robust classification tasks across diverse datasets. PILC-BSCSO’s superior performance can be attributed to several factors: enhanced exploration and exploitation, population initialization, and fitness function evaluation. PILC-BSCSO leverages the Pinhole-Imaging Opposition-Based Learning (PIOBL) scheme and the crossover operator to enhance both the exploration and exploitation phases. This allows it to effectively explore a wide solution space while also exploiting promising regions more efficiently, leading to improved solutions. The algorithm also uses an initial population strategy based on differential expression analysis. This strategy provides a better-initialized population, guiding the optimization process toward more promising solutions from the start. It also employs repeated 10-fold cross-validation with three repetitions contributing to more stable and reliable results, especially when dealing with unbalanced datasets. Additionally, utilizing the kappa measure of SVM further enhances the appropriateness of the evaluation metric for accurately assessing model performance in the context of class imbalance. This approach ensures a robust evaluation framework that is well-suited for the challenges posed by the dataset at hand.

### 3.3. Experimental Results on Liver Hepatocellular Carcinoma TCGA

To demonstrate the effectiveness of the proposed method, it was applied to data on HCC sourced from TCGA. HCC, a devastating malignancy ranked as the third leading cause of global cancer-related deaths, often evades early detection, resulting in diagnosis at advanced stages. Therefore, the development of innovative treatment targets is of paramount importance to enhance patient survival outcomes.

The RNA-Seq data encompassed 371 samples from HCC patients and 50 control samples, all derived from the TCGA-liver hepatocellular carcinoma (LIHC) dataset, comprising a total of 421 samples and 56,602 genes. Following data acquisition, various preprocessing steps were executed, including the removal of genes with low counts, conversion of counts to DGEList format, quality control, and normalization to mitigate batch effects. Subsequently, 1656 genes with |LogFC| > 2 were identified as DEGs out of the initial 14,899 genes, based on an adjusted *p*-value threshold of 0.05 (as depicted in Figure 3). The dataset was partitioned into training (75%) and testing (25%) sets, with the latter serving as an independent dataset to validate the PILC-BSCSO results.

Figure 4 illustrates the convergence behavior of the TCGA-LIHC training dataset comprising 1546 DEGs and 317 samples for both BSCSO and PILC-BSCSO. The experimental results on the test data (104 samples) reveal that PILC-BSCSO outperforms BSCSO in terms of classification accuracy, achieving an average of 98.87% ± 1.2, compared to BSCSO’s 97.6% ± 3. PILC-BSCSO demonstrates superior efficiency in feature selection, with an average selection of 8 ± 2.6 genes, in contrast to BSCSO’s average of 73 ± 20.2 genes, for the achievement of higher classification accuracy.

Figure 5 portrays the expression patterns of the best subset of identified genes, including ANGPTL6 [28], HMMR [29], CHST4 [30], COL15A1 [31], and PZP [32], utilizing the proposed approach. These genes exhibit remarkable classification accuracy and an Area Under the Curve (AUC) of 100% in the test data.

**Figure 7:**
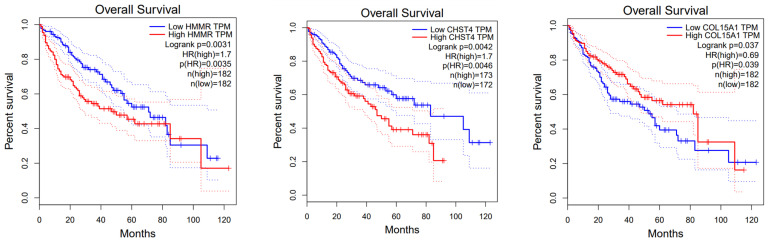
Kaplan–Meier analysis of the survival rates of the high- and low-expression groups of HMMR, CHST4, and COL15A1.

Furthermore, Kaplan–Meier survival analyses were conducted to evaluate the prognostic potential of these genes. Among the five genes in the subset, HMMR, CHST4, and COL15A1 emerged as potential independent biomarkers (Figure 7), signifying a robust and statistically significant association with patient survival in HCC.

**Figure 8:**
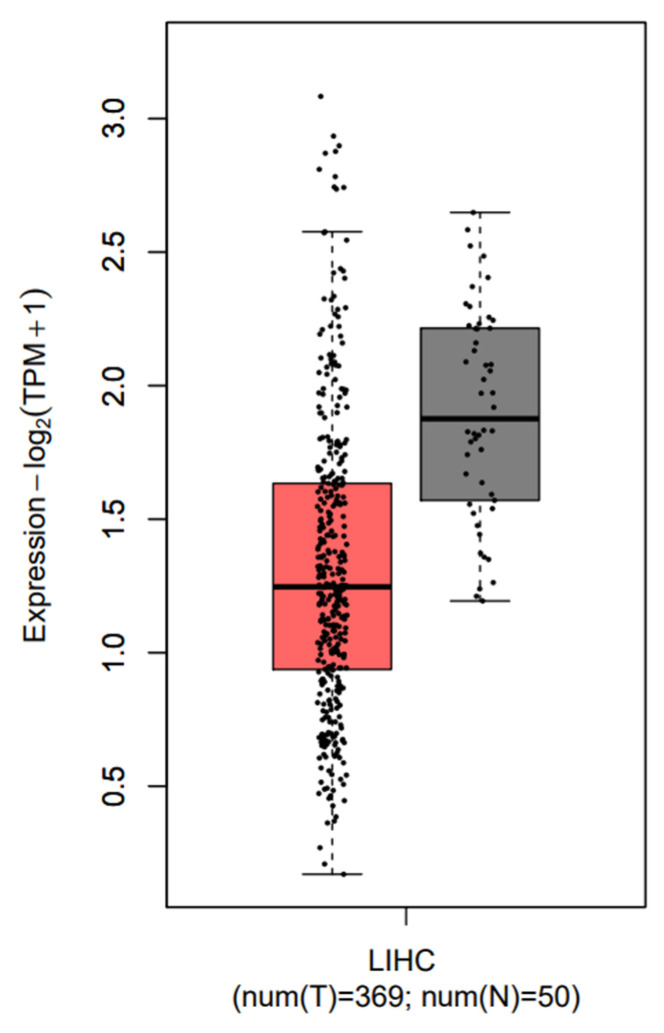
Tissue-wise expression patterns of the identified best subset of genes associated with LIHC. Red color indicates tumors, and gray indicates normal samples.

Figure 8 depicts the tissue-wise expression patterns of the identified best subset of genes associated with LIHC. From this figure, it can be observed that the identified subset of five genes (ANGPTL6, HMMR, CHST4, COL15A1, and PZP) has discriminative gene expression patterns. These genes can potentially serve as diagnostic or prognostic biomarkers, aiding in the early detection or risk assessment of LIHC.

## 4. Discussion

Due to the rapid technological improvement in medical research, a vast volume of biomedical data is regularly created from various biomedical equipment and investigations these days. The effective analysis of this biomedical data, such as identifying the key biological and diagnostic features, is a difficult challenge. Here, a new feature selection method based on the BSCSO algorithm was proposed. Pinhole-imaging-based learning strategy and crossover operator are combined with BSCSO to design the PILC-BSCSO algorithm which is capable of efficiently addressing feature selection problems for high-dimensional biomedical data. Experimental results on three benchmark datasets reveal that the suggested PILC-BSCSO-SVM method can achieve a superior classification accuracy with a lower number of features simultaneously when compared to the 11 most recent state-of-the-art methods. In the context of HCC analysis, the PILC-BSCSO algorithm demonstrated outstanding performance. It successfully pinpointed a subset of target genes, including HMMR, CHST4, and COL15A1, that function as both prognostic and diagnostic biomarkers. The proposed approach holds promise for enhancing HCC diagnosis and patient outcome prediction. 

While the PILC-BSCSO algorithm shows promise, it is important to acknowledge potential limitations, including the need for further validation in larger and more diverse datasets such as single-cell data to ensure its generalizability. Although PILC-BSCSO demonstrates impressive feature selection and classification accuracy, the algorithm’s output may lack interpretability, particularly when dealing with a very large number of genes. Identifying the biological relevance of the selected genes or understanding the underlying biological mechanisms contributing to high classification accuracy may require additional post-processing and domain expertise. Enhancing the algorithm’s interpretability and providing insights into the biological significance of the selected genes could be an area for further improvement. The robustness of PILC-BSCSO in selecting biologically informative genes can indeed be a potential concern, as it is for many feature selection algorithms.

In future work, other transfer functions, such as X-shaped and U-shaped, might be used to determine how they affect the suggested approach. Additionally, we believe that the incorporation of Protein–Protein interaction networks will improve the algorithm’s capacity for biomarker identification. Furthermore, the suggested PILC-BSCSO may be evaluated to address various optimization issues, including clustering, task scheduling in fog computing, image segmentation, sentiment analysis, and more. PILC-BSCSO can be adapted to tackle clustering tasks by modifying its objective function and fitness evaluation criteria. Instead of feature selection, the algorithm could be tailored to group similar data points together while maximizing the dissimilarity between clusters. The algorithm’s optimization capabilities can help identify meaningful cluster centroids or representative data points, contributing to improved clustering accuracy and robustness. By defining a suitable objective function, PILC-BSCSO may be applied to task scheduling in fog computing. The method can efficiently schedule jobs to fog nodes, minimizing execution time and resource usage while optimizing overall system performance. In image processing, PILC-BSCSO can be adapted for image segmentation tasks. The objective function can be designed to identify optimal segmentation boundaries within an image. The algorithm’s optimization capabilities can help automate the process of partitioning an image into distinct regions or objects based on various image attributes, such as intensity, color, or texture. PILC-BSCSO can contribute to sentiment analysis by optimizing feature selection for sentiment classification tasks. The algorithm can identify the most informative features from text or data sources, enhancing the performance of sentiment analysis models. In each of these applications, the key lies in customizing the objective function, fitness evaluation criteria, and problem-specific parameters to align with the optimization goals. PILC-BSCSO’s adaptability and optimization capabilities make it a versatile tool for addressing a wide range of optimization challenges beyond gene selection, enhancing performance and efficiency in diverse domains.

In summary, PILC-BSCSO holds the potential to significantly impact the field of biomedicine by providing an advanced gene selection approach that enhances disease diagnosis and prognosis, and its versatility extends to broader applications in various domains, including healthcare, bioinformatics, and beyond.

## Figures and Tables

**Table 1:** Characteristics of Gene Expression Datasets.

Dataset Name	No. of Samples	No. of Features	No. of Classes	Distribution of Class Label
Colon cancer	62	2000	2	40, 22
CNS	60	7129	2	39, 21
Breast	97	24,481	2	51, 46
TCGA-LIHC	421	56,602	2	371, 50

**Table 2:** The LOOCV classification accuracy of identified DEGs, mRMR, and MCFS with an SVM classifier.

Dataset Name	All Features	DEGs	mRMR (50)	mRMR (100)	mRMR (200)	mRMR (300)	MCFS (50)	MCFS (100)	MCFS (200)	MCFS (300)
Colon cancer	83.87	85.48	80.64	83.87	83.87	80.64	79.03	88.70	85.483	88.70
CNS	68.33	90	60	6333	7833	68.33	81.66	0.85	91.66	93.33
Breast	67.01	75.25	76.28	78.35	78.35	79.38	72.16	76.28	87.62	89.69

**Table 3:** Comparison between BSCSO and PILC-BSCSO in terms of classification accuracy and number of selected genes.

Dataset	Metrics	Accuracy				#Genes			
		BSCSO	GA	PSO	PILC-BSCSO	BSCSO	GA	PSO	PILC-BSCSO
Colon	AVG	97.63	91.311	94.35	99.22	8.33	133.6	70.4	15
	best	100	93.54	98.38	100	6	113	50	10
	worst	93.81	85	83.87	96.9	9	145	89	23
	STDEV	2.35	3.349	4.47	1.348	1.966	12.91	15.51	5.244
	*t*-test (*p*-value)	0.0195	0.0066	0.0519		0.0159	1.2259 × 10⁻⁵	0.0022	
CNS	AVG	99.34	98.332	99.16	100	33.25	100.5	73.4	16.25
	best	100	100	100	100	14	45	54	13
	worst	98.49	95	98.333	100	59	144	90	22
	STDEV	0.755	2.041	0.914	0	18.76	42.914	13.29	4.0311
	*t*-test (*p*-value)	0.07198	0.0622	0.0755		0.0479	0.00808	0.00118	
Breast	AVG	91.819	91.06	96.2	96.38	11.4	62	58	26.4
	best	97.926	95.87	100	100	5	56	52	15
	worst	88.7533	84.53	93.81	93.98	16	66	65	40
	STDEV	3.808	4.36	2.61	2.5	4.722	4.32	5.09	12.30
	*t*-test (*p*-value)	0.00097	0.008246	0.6330		0.00730	0.00036	0.00047	

Note: ‘#’ represents number of selected genes.

**Table 4:** Comparing the performance of the suggested methodology to approaches from the literature.

Methods	High Dimensional Biomedical Datasets					
	Colon Cancer		CNS		Breast	
	∣#G∣	ACC	∣#G∣	ACC	∣#G∣	ACC
PILC-BSCSO	15	99.22	16.25	100	26.4	96.38
BMSCSO [2]	997.80	93.33	-	-	-	-
mRMR-MBAO [5]	16.11	95.74	21.37	88.57	23.58	89.12
SU-RSHSA [10]	7.59	93.17	13.15	89.36	18.31	80.40
mRMR-DBH [9]	12	97.02	39.75	97.19	14	90.21
IBCFPA [7]	25.90	92.16	25.2	84.82	-	-
MIM-MFO [6]	24.25	99.19	17	85.00	22.50	84.11
BCROSAT [1]	20.5	92.31	21.40	82.00	-	-
ISFLA [8]	37.1	89.56	41.1	77.46	-	-
TOPSIS-Jaya [11]	18.90	97.76	8.7	96.22	-	-
IG-MBKH [12]	17.10	96.47	14.70	90.34	-	-
mRMR-BCOOT-CSA [13]	8.75	94.75	7	93.22	15	95.54

Note: ‘#’ represents number of selected genes.

## Data Availability

All relevant data are within the paper.
